# Association between high-dose erythropoiesis-stimulating agents, inflammatory biomarkers, and soluble erythropoietin receptors

**DOI:** 10.1186/1471-2369-12-67

**Published:** 2011-12-12

**Authors:** Jula K Inrig, Suzanne K Bryskin, Uptal D Patel, Murat Arcasoy, Lynda A Szczech

**Affiliations:** 1 Department of Medicine, University of Texas Southwestern Medical Center, 5323 Harry Hines Blvd, Dallas, Texas, 75390-8523, USA; 2 Department of Medicine, Duke University Medical Center, 2301 Erwin Road, Durham, North Carolina, 27708, USA; 3 Department of Medicine, Duke Clinical Research Institute, 2400 Pratt Street, Durham, North Carolina, 27705, USA

## Abstract

**Background:**

High-dose erythropoiesis-stimulating agents (ESA) for anemia of chronic kidney disease (CKD) have been associated with adverse clinical outcomes and do not always improve erythropoiesis. We hypothesized that high-dose ESA requirement would be associated with elevated inflammatory biomarkers, decreased adipokines, and increased circulating, endogenous soluble erythropoietin receptors (sEpoR).

**Methods:**

A cross-sectional cohort of anemic 32 CKD participants receiving ESA were enrolled at a single center and cytokine profiles, adipokines, and sEpoR were compared between participants stratified by ESA dose requirement (usual-dose darbepoetin-α (< 1 μg/kg/week) and high-dose (≥1 μg/kg/week)).

**Results:**

Baseline characteristics were similar between groups; however, hemoglobin was lower among participants on high-dose (1.4 μg/kg/week) vs usual-dose (0.5 μg/kg/week) ESA.

In adjusted analyses, high-dose ESA was associated with an increased odds for elevations in c-reactive protein and interleukin-6 (p < 0.05 for both). There was no correlation between high-dose ESA and adipokines. Higher ESA dose correlated with higher levels of sEpoR (r_s _= 0.39, p = 0.03). In adjusted analyses, higher ESA dose (per μcg/kg/week) was associated with a 53% greater odds of sEpoR being above the median (p < 0.05).

**Conclusion:**

High-dose ESA requirement among anemic CKD participants was associated with elevated inflammatory biomarkers and higher levels of circulating sEpoR, an inhibitor of erythropoiesis. Further research confirming these findings is warranted.

**Trial registration:**

Clinicaltrials.gov NCT00526747

## Background

Clinical trials in patients with chronic kidney disease (CKD) have demonstrated that attempting to achieve a higher hemoglobin with erythropoiesis-stimulating agents (ESA) leads to adverse cardiovascular outcomes [1-4]. Recently, secondary analyses have suggested the increased cardiovascular morbidity and mortality among anemic CKD participants randomized to higher hemoglobin targets may be partially explained by the use of high-doses of ESA [5,6]. While it has been suggested that ESA therapy may be proinflammatory [7] or have direct toxic effects on the cardiovascular system [8,9], the underlying mechanism of the elevated cardiovascular risk associated with high-dose ESA has yet to be elucidated.

Inflammation and malnutrition among patients with CKD have been demonstrated to be significant contributors to accelerated atherosclerosis and increased cardiovascular mortality [10-13], and may contribute to the excess risk associated with high-dose ESA. Although elevations of select inflammatory cytokines have been demonstrated in anemic CKD patients treated with ESA and in CKD patients overtly resistant to ESA therapy [14,15], a clear dose relationship has not been demonstrated. Further, chronic inflammation can lead to protein energy wasting which may mediate another pathway for adverse cardiovascular outcomes in CKD patients [16,17]. As a result, persistent elevations in inflammatory cytokines associated with high-dose ESA may also be associated with adipokine dysregulation.

In addition to a potential association with inflammation and malnutrition, increasing ESA requirement and dosing does not always result in improved hemoglobin levels, the reasons for which are unclear. Erythropoietin stimulates erythropoiesis by binding to its cell surface receptor EpoR - a member of the cytokine receptor family that is expressed on erythroid cells in the bone marrow. An alternatively spliced mRNA isoform of EpoR giving rise to a soluble form (sEpoR) that lacks the transmembrane domain of the receptor and potentially secreted into extracellular space has been described [18-21]. A recombinant form of human sEpoR has been shown to bind Epo with high affinity and act as a potent Epo antagonist in diverse in vitro and in vivo experimental models [22-25]. Endogenous sEpoR has been detected in human serum and plasma [26-29] but its physiologic role and biologic activity have not been defined. Whether endogenous sEpoR may modulate the erythropoietic response to ESA therapy is not known and the relationship between circulating sEpoR levels and ESA dosing in anemic patients with CKD remains to be characterized.

We hypothesized that the requirement for high-dose ESA therapy in some anemic CKD patients may be associated with elevated inflammatory markers and/or impaired nutritional status. Finding such an association may provide an insight into possible mechanisms for the adverse outcomes associated with high-dose ESA administration. Secondly, we hypothesized that there may be a direct relationship between increasing ESA dose requirement and higher levels of circulating sEpoR. To address these questions, this study compared demographic factors, C-reactive protein (CRP), cytokine profiles, adiponectin, leptin, and plasma soluble erythropoietin receptor levels in a cross-sectional cohort of anemic CKD participants who required treatment with either high-doses or usual-doses of ESA.

## Methods

### Patients

The study sample is a prospective cohort of anemic CKD patients treated with ESA therapy enrolled from the Durham Nephrology anemia clinic, a private practice in Durham, North Carolina. Inclusion criteria included: ≥ 18 years of age, CKD defined as an estimated glomerular filtration rate < 60 ml/min/1.73 m^2 ^using the Modification of Diet in Renal Disease Study II equation (eGFR (ml/min per 1.73 m^2^) = 186 × [Cr (mg/100 ml)]^-1.154 ^x (age)^-0.203 ^x (1.21 if subject is black)), active treatment with darbepoetin-α therapy, and anemia deemed secondary to chronic kidney disease. Exclusion criteria included: an active gastrointestinal (GI) bleed or history of GI bleed within the prior 3 months, uncontrolled hyperparathyroidism (parathyroid hormone > 550), untreated vitamin B12 or folate deficiency, untreated iron deficiency (transferrin saturation < 20% and ferritin < 100), overt infection, active hemolysis, hemoglobinopathies, known adverse response to ESA, prior kidney transplant or aluminum toxicity.

Patients were screened for enrollment and all patients requiring high-dose ESA therapy over the prior 3 months (defined *a priori *as receiving ≥ 1 mcg/kg/week of darbepoetin-α) and a random sample of patients requiring usual-dose ESA therapy (defined *a priori *as < 1 mcg/kg/week darbepoetin-α) were screened and enrolled between September 1, 2007 and April 31, 2008. Patients were identified by record review. On the day of the patient's appointment in the anemia management clinic, patients were approached by study personnel to ask if they were interested in participating in the study. If the patients agreed to participate, they were asked to sign informed consent and a HIPAA form. Following informed consent, the following data were collected (through a one-on one participant interview and chart review): age, weight, ESA doses during the previous 3 months, dosing interval, blood pressure, heart rate, past medical history, and medications. Laboratory measures including serum albumin, hemoglobin, iron status, serum chemistry, and parathyroid hormone (analyzed at one central laboratory) were collected as part of routine patient care and were retrieved through chart review. After the interview, the participant was scheduled for a 5 ml blood draw prior to the administration of the next ESA dose at the subsequent anemia clinic visit. All blood samples were immediately processed and stored at -80°C until analysis.

The study was approved by the Duke University Medical Center Institutional Review Board and was registered with clinicaltrials.gov (NCT00526747).

### Biochemical analyses

Utilizing multiplex protein array technology (Thermo Fisher Scientific, Massachusetts, USA), the plasma was assayed for the following cytokines: interferon-γ, TNF-α, IL-1β, IL-2, IL-6, IL-8, IL-10, IL-12p40 and IL-12p70 [30]. Details are available in Additional File 1. All measurements were run in duplicate and the laboratory technicians were blinded to the clinical characteristics of the subjects being studied.

C-reactive protein was determined by a commercially available highly sensitive latex-based immunoassay and quantitatively measured using an immunoturbidimetric assay on the Hitachi autoanalyzer (Roche Diagnostics, Indianapolis, USA).

Adiponectin and circulating leptin concentrations were analyzed by radioimmunoassay (Linco Research, Inc, St Louis, MO, USA) which has been previously described and validated [31]. The assays employ a polyclonal (rabbit) antibody raised against recombinant human adiponectin and leptin, respectively. The gamma counter used for these measurements was a Perkin-Elmer Wallac Wizard 1470.

For quantification of sEpoR in plasma, samples were analyzed via ELISA (R&D Systems, Minneapolis, MN). Details are available in Additional File 2.

### Statistical analysis

Continuous data are presented as means (standard deviations) or medians (25-75% interquartile range) and compared with the Satterthwaite two sample t-test or Wilcoxon rank sum test. Categorical data are presented as counts and percents and compared with the chi^2 ^statistic or Fisher's exact test.

Given the non-normal distribution of cytokines and adipokines, markers were divided into quartiles and logistic regression was performed to calculate an odds ratio (case-control relative risk) for having a biomarker above/below the median among participants on high-dose ESA vs normal-dose ESA, as previously described [7]. In adjusted analyses of predictors of inflammatory cytokines, models were controlled for gender, hemoglobin, and ferritin. A graphical bar plot was created for the odds (5-95% CI) of cytokines being above the median among participants on high vs usual dose ESA. In adjusted analyses of predictors of adipokines, models were adjusted for gender, hemoglobin and weight.

Analyses of sEpoR were performed similarly. To graphically describe the relationship between ESA dose and sEpoR, a scatter plot with a linear regression line (95% confidence intervals of the predicted mean) was generated. In adjusted analyses of predictors of sEpoR, the model was adjusted for gender, hemoglobin, eGFR, and ESA dose.

All data was analyzed using SAS Eguide 4.1 (SAS Institute, North Carolina, USA).

## Results

### Participant Characteristics by ESA Dose

A total of 11 participants requiring high-dose ESA therapy (≥ 1 mcg/kg/wk of darbepoetin-α) and 21 participants treated with usual-dose ESA therapy (< 1 mcg/kg/wk of darbepoetin-α) were enrolled into the study. Demographics, baseline characteristics, and medications were similar between groups (Table 1). While most laboratory parameters were similar between groups, the 3-month average hemoglobin was 10.4 g/dl in the high-dose ESA group compared to 11.5 g/dl in the usual-dose ESA (p = 0.0005).

**Table 1:** Demographics and baseline characteristics of the cohort of anemic chronic kidney disease participants treated with usual or high-dose ESA

Variable	High-dose ESA (≥1 mcg/kg/wk of darbepoetin-α) (n = 11)	Usual-dose ESA (< 1 mcg/kg/wk of darbepoetin-α) (n = 21)	P-value
**Age**	71.1 ± 11.2	67.4 ± 10.2	0.38

**Primary Cause of Kidney Disease (%)**			0.13
Diabetes	3 (27.3%)	10 (47.6%)	
Hypertension	3 (27.3%)	6 (28.6%)	
Glomerulonephritis	0 (0.0%)	2 (9.5%)	
Unknown	3 (27.3%)	0 (0.0%)	
Other	2 (18.2%)	3 (14.3%)	

**Male Sex**	1 (9%)	8 (38.1%)	0.11

**Race (%)**			0.71
Black	5 (45.5%)	12 (57.1%)	
White	6 (54.6%)	9 (42.9%)	

**Weight (kg)**	80.6 ± 29.6	92.0 ± 21.8	0.28

**Blood Pressure (mmHg)**			
Systolic	127.0 ± 11.0	130.0 ± 16.2	0.54
Diastolic	67.2 ± 8.3	68.8 ± 13.1	0.72

**Heart rate (beats/min)**	80.4 ± 19.4	74.3 ± 11.2	0.36

**Baseline Comorbidities (%)**			
Diabetes, non insulin requiring	3 (27.3%)	7 (33.3%)	0.99
Diabetes, insulin requiring	5 (45.5%)	9 (42.9%)	0.99
Cerebrovascular accident	0 (0.0%)	3 (14.3%)	0.53
Hypertension	11 (100.0%)	21 (100.0%)	0.99
Hyperparathyroidism	7 (63.6%)	15 (71.4%)	0.70
Congestive heart failure	2 (18.2%)	1 (4.8%)	0.27
Cancer	0 (0.0%)	2 (9.5%)	0.53
Coronary artery disease	3 (27.3%)	6 (28.6%)	0.99

**Darbepoetin-α dose **(3-month average)	94.1 mcg/week (1.4 mcg/kg/week)	49.7 mcg/week (0.5 mcg/kg/week)	0.001

**Baseline Medications (%)**			
Vitamin D	7 (63.6%)	14 (66.7%)	0.99
HMG-CoA reductase inhibitor	7 (63.6%)	10 (47.6%)	0.47
Oral Iron	7 (63.6%)	17 (81.0%)	0.40
Intravenous Iron	1 (9.1%)	1 (4.8%)	0.99
Non-calcium containing phosphorus binder	0 (0.0%)	1 (4.8%)	0.99
Calcium-containing phosphorus binder	2 (18.2%)	3 (14.3%)	0.99
Coumadin	2 (18.2%)	1 (4.8%)	0.27
Aspirin	6 (54.6%)	14 (66.7%)	0.70
ACE/ARB	7 (63.6%)	16 (76.2%)	0.68
Alpha antagonist	0 (0.0%)	4 (19.1%)	0.27
Beta blocker	6 (54.6%)	13 (61.9%)	0.72
Calcium channel blocker	5 (45.5%)	10 (47.6%)	0.99
Nitroglycerin	1 (9.1%)	3 (14.3%)	0.99

**Hematology Laboratory Values**			
3-month average hemoglobin (g/dl)	10.4 ± 0.7	11.5 ± 0.8	0.0005
Hemoglobin at enrollment (g/dl)	10.8 ± 1.0	12.3 ± 0.9	0.0004
Serum Iron (mcg/dl)	75.9 ± 31.6	78.1 ± 21.8	0.94
Serum Ferritin (ng/ml, median, IQR)	343.0 (224.0, 673.0)	353.0 (314.0, 493.0)	0.93*
Transferrin saturation, %	28.9 ± 10.3	32.1 ± 8.6	0.59

**Chemistry Laboratory Values**			
Serum blood urea nitrogen (mg/dl)	58.3 ± 24.8	54.6 ± 14.8	0.66
Serum creatinine (mg/dl)	2.6 ± 1.2	2.7 ± 1.1	0.88
MDRD GFR (ml/min per 1.73 m^2^)	25.9 ± 15.1	25.9 ± 9.3	0.99
Serum glucose (mg/dl, median, IQR)	109.0 (88.0, 134.0)	113.0 (89.0, 154.5)	0.47*
Serum albumin (mg/dl)	4.0 ± 0.4	3.9 ± 0.3	0.38
Serum phosphorus (mg/dl)	4.5 ± 1.0	4.1 ± 0.6	0.22
Serum PTH (pg ml^-1^, median, IQR)	148.5 (39.0, 208.0)	99.0 (52.0, 167.0)	0.59*

*nonparametric test used

### ESA dose and inflammatory biomarkers

The median levels (pg/ml) of inflammatory biomarkers among participants on high-dose and usual-dose ESA are shown in Table 2. The unadjusted odds ratio for participants on high-dose ESA and having an inflammatory biomarker above the median were compared with participants on usual-dose ESA and having an inflammatory biomarker above the median (Table 2). In all, participants on high-dose ESA (vs usual-dose ESA) were more likely to exhibit inflammatory biomarkers above the median, but only CRP was statistically significant.

**Table 2:** Median cytokine levels among participants treated with high-dose and usual-dose ESA and unadjusted and adjusted odds of cytokine being above the median

Variable	High-dose ESA (≥1 mcg/kg/wk of darbepoetin-α) (n = 11)	Usual-Dose ESA (< 1 mcg/kg/wk of darbepoetin-α) (n = 21)	Unadjusted Odds Ratio, * (5-95% CI) p-value	Adjusted Odds Ratio**, (5-95% CI) p-value
C-reactive protein (mg/l)	7.7 (4.8, 9.7)	2.4 (1.1, 9.2)	9.0 (1.5-53.4) p = 0.01	16.0 (1.5-174.5) p = 0.02

Interferon-γ (pg/mL)	12.4 (4.4, 18.3)	8.5 (3.8, 11.2)	4.3 (0.88-21.3) p = 0.06	4.5 (0.5-37.2) p = 0.2

Tumor necrosis factor-α (pg/mL)	68.7 (10.3, 202.4)	41.2 (15.8, 65.6)	1.32 (0.31-5.7) p = 0.7	1.7 (0.2-14.4) p = 0.6

Interleukin-1β (pg/mL)	1.0 (0.2, 2.0)	0.6 (0.3, 1.6)	1.32 (0.31-5.7) p = 0.7	1.3 (0.1-11.4) p = 0.8

Interleukin-2 (pg/mL)	18.2 (6.3, 33.1)	9.2 (4.5, 22.2)	4.3 (0.88-21.3) p = 0.06	2.4 (0.3-18.8) p = 0.4

Interleukin-6 (pg/mL)	21.3 (17.3, 26.3)	16.2 (13.5, 21.9)	4.3 (0.88-21.3) p = 0.06	12.3 (1.1-143.2) p = 0.04

Interleukin-8 (pg/mL)	63.5 (33.9, 63.5)	44.5 (34.3, 65.0)	1.32 (0.31-5.7) p = 0.7	1.1 (0.2-8.3), p = 0.9

Interleukin-10 (pg/mL)	4.2 (1.3, 7.6)	1.5 (1.0, 3.3)	4.3 (0.88-21.3) p = 0.06	4.3 (0.5-35.0) p = 0.2

IL-12p40 (pg/mL)	32.8 (15.9, 37.0)	22.8 (16.1, 32.2)	2.3 (0.52-10.5) p = 0.45	3.1 (0.4-23.2) p = 0.3

IL-12p70 (pg/mL)	4.4 (1.1, 8.1)	2.7 (1.6, 4.0)	4.3 (0.88-21.3) p = 0.06	5.6 (0.7-47.2) p = 0.1

Median and 25^th ^to 75^th ^interquartile range

*Odds ratio of cytokine being above the median among those in high vs usual dose ESA,

**Adjusted for gender, hemoglobin and ferritin

**Figure 1:**
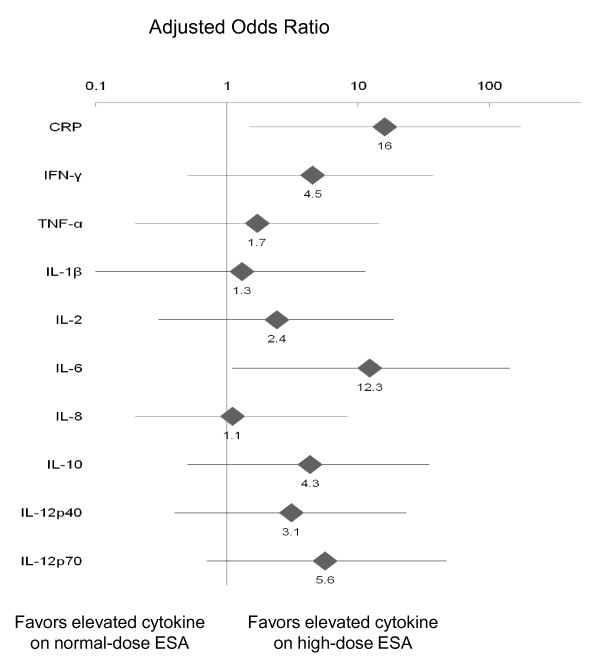
**Adjusted odds ratio for elevated inflammatory cytokines associated with high-dose vs usual-dose ESA therapy**. Odds ratios were adjusted for gender, hemoglobin, and ferritin.

After adjusting for gender, hemoglobin, and ferritin, high-dose ESA therapy was independently associated with an increased odds ratio of CRP and of IL-6 being above the median (Table 2, Figure 1). While not statistically significant, all other inflammatory cytokines tested (including IFN-γ, TNF-α, IL-1β, IL-2, IL-8, IL-10, IL-12p40, and IL-12p70) were more likely to be above the median among participants on high-dose ESA compared to usual-dose ESA.

### ESA dose and adipokines

The median level of adiponectin (ng/ml) was 18, 718.6 among participants on usual-dose ESA and 15, 397.4 among participants on high-dose ESA (Table 3). Given the inverse relationship between adiponectin and clinical outcomes [32], we tested whether participants on high-dose ESA were more likely to have an adiponectin level below the median. In unadjusted analyses, there was no difference between groups in odds of adiponectin being below the medians; however, in adjusted analyses there were trends towards lower odds of adiponectin being below the median among those on high-dose vs usual dose ESA (Table 3).

**Table 3:** Median adipokine levels among participants treated with high-dose and usual-dose ESA and unadjusted and adjusted odds of adipokine being below the median

Variable	High-dose ESA (≥1 mcg/kg/wk of darbepoetin-α) (n = 11)	Usual-Dose ESA (< 1 mcg/kg/wk of darbepoetin-α) (n = 21)	Unadjusted Odds Ratio, * (5-95% CI) p-value	Adjusted Odds Ratio, * (5-95% CI) p-value
Adiponectin (ng/ml)	18718.6 (13328.6, 33469.4)	15397.4 (8032.5, 53063.7)	0.76 (0.2-3.3) p = 0.7	7.8 (0.5-109.2) p = 0.1

Leptin (ng/ml)	30.9 (1.5, 102.6)	43.4 (9.8, 82.6)	2.33 (0.5-10.5) p = 0.3	1.7 (0.1-19.2) p = 0.7

*Odds of cytokine being below the median among those in high vs usual dose ESA,

**Adjusted for gender, hemoglobin, and weight

The median level of leptin (ng/ml) was 30.9 and 43.4 among participants on high-dose and usual-dose ESA, respectively (Table 3). Neither unadjusted nor adjusted analyses identified significant differences in odds of leptin levels being below the median between groups.

### ESA dose and Soluble Epo Receptor

The median level of soluble Epo receptor (sEpoR) was 2.5 pg/ml among participants on high-dose ESA and 0.7 pg/ml among participants on usual-dose ESA. In unadjusted analyses, 63.6% of participants on high-dose ESA were above the median of sEpoR compared to 42.9% of participants on usual-dose ESA.

**Figure 2:**
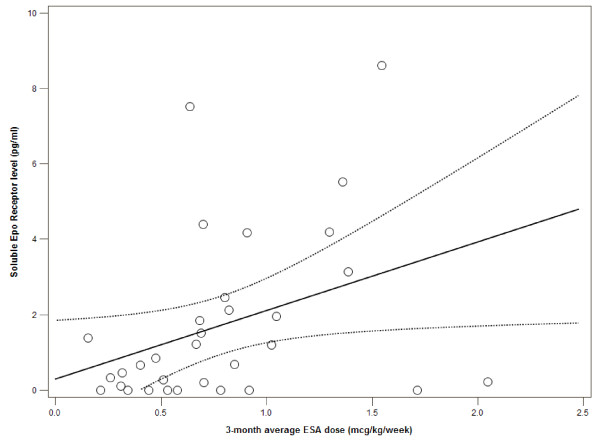
**Relationship between soluble Epo Receptor level and ESA dose**. Scatter plot with linear regression line (Y = β_0_+β_1_X) and 95% confidence intervals of the mean predicted value of soluble Epo Receptor.

By the Spearman's rank correlation test, there was a modest correlation between 3-month average ESA dose (in mcg/kg/week) and sEpoR (r_s _= 0.39, p = 0.03). The relationship between ESA dose and sEpoR can be seen graphically in Figure 2. Considering this relationship may have been partially explained by differences in hemoglobin, we tested whether there was a correlation between hemoglobin and sEpoR, however no significant relationship was identified (r_s _= -0.20, p = 0.3). There was also no correlation between any of the aforementioned cytokines and sEpoR (data not shown). We also performed logistic regression to determine if average ESA dose (in mcg/kg/week) remained independently associated with a sEpoR level above the median. In adjusted analyses, higher ESA dose (per mcg/kg/week increase) was significantly associated with a greater odds of sEpoR being above the median (OR 1.53, CI 1.03-2.3, p = 0.04).

## Discussion

Anemia of chronic kidney disease is common, yet the optimal treatment to improve outcomes remains uncertain. In light of recent analyses demonstrating high-dose ESA therapy to be associated with adverse outcomes [4-6], our study sought to describe biochemical alterations which may be associated with high-dose ESA. Among participants treated with higher doses of ESA, we identified significantly higher levels of inflammatory markers CRP and IL-6, which could be in the causal pathway for adverse outcomes associated with high-dose ESA. Second, our study is the first to describe *in vivo *a direct relationship between ESA dose and soluble Epo receptor in CKD patients as a potential modulator of erythropoietic response to ESA therapy. However, given the observational nature of our study, further studies are required to confirm these findings.

### ESA dose and inflammation

Although the association between chronic kidney disease and elevated inflammatory biomarkers has been well established, the relationship between CKD-related anemia, inflammation/oxidative stress and the use of ESAs has only recently been described [7,15,33]. A recent study by Keithi-Reddy et al demonstrated that CKD-related anemia is not only associated with elevated inflammatory cytokines but patients with CKD-related anemia treated with ESA also have higher TNF-α and IL-6 levels compared to ESA-naïve anemic CKD patients [7]. Other studies in CKD patients have demonstrated ESA resistance to be associated with elevated inflammatory cytokines, including IL-6, IFN-γ, and TNF-α[34-37]. Our study extends these prior observations by demonstrating that patients who are administered higher ESA doses (even if not overtly ESA resistant) also exhibit higher levels of inflammatory biomarkers, including IL-6 and CRP. Given our small sample size and the short duration of our study, further prospective studies will need to define whether changes in ESA dose effect the levels of inflammatory biomarkers over time.

### ESA dose and adipokines

Adverse outcomes related to chronically elevated inflammatory biomarkers have in part been explained by the role of cytokines in the development of the protein-energy wasting [38-40]. In our study we compared plasma adiponectin and leptin levels in participants treated with usual and high-dose ESA. Despite differences in IL-6 and CRP which have been associated with protein-energy wasting, we found no significant relationship between high-dose ESA dose and low levels of adiponectin or leptin. Our failure to find a difference between participants may be due to a number of factors. First, development of nutritional dysregulation related to inflammation likely occurs over longer periods than observed in our short study. Second, our cohort was a healthier group of CKD participants separate and distinct from hemodialysis patients in which protein energy wasting is more prevalent. Finally, our small sample size, may have limited our discriminatory capability to identify significant differences.

### ESA dose and soluble EPO receptor

In this study, we postulated that circulating plasma sEpor levels may be relatively elevated in CKD patients requiring high doses of ESA for the treatment of anemia and found that higher ESA dose requirement was correlated with higher levels of circulating sEpoR. Unlike one prior study [29], we found no relationship between other measured cytokines and plasma sEpoR level. We also found no relationship between hemoglobin level and sEpoR suggesting severity of anemia may not correlate with sEpoR levels, consistent with the findings of previous studies [26,27,41]. Although a recombinant form of sEpoR was shown to act as an antagonist of Epo in in vitro and in vivo studies of Epo-EpoR biology and function, it is not known whether low levels of endogenous, circulating plasma sEpoR can directly modulate erythropoietic responses to exogenous ESAs. While this study is the first to assess the relationship between ESA dose and sEpoR level among CKD patients, one previous study which measured sEpoR levels in patients with liver and kidney disease [28] reported elevated sEpoR levels in the plasma of five hemodialysis patients on EPO when compared to healthy controls. In the current study, we did not measure pre-ESA plasma sEpoR levels and therefore cannot determine whether ESA therapy itself may be involved in upregulation of sEpoR level in a dose-dependent manner. An alternative possibility is that higher baseline sEpoR levels prior to ESA therapy initiation may serve as a predictor of ESA response and dose requirement. In fact, a recent retrospective analysis among incident hemodialysis patients identified higher sEpoR levels at dialysis initiation to be associated with higher ESA requirements [29]. Further studies are needed to characterize the relationship between endogenous sEpoR and ESA responses and to elucidate the physiologic role of sEpoR on regulation and control of erythropoiesis.

### Limitations

Our study has several limitations. It was an observational cohort and causality cannot be determined, thus our results should be considered hypothesis generating. Second, our study measured biomarkers and sEpoR levels at one point in time in patients already on ESA therapy; thus we cannot determine the relationship between changes in ESA and changes in these parameters over the course of therapy or prior to administration of ESA. Prospective studies which measure biomarkers over time to determine the natural course and changes in these biomarkers in individual patients could help further elucidate whether our findings are due to patient-specific factors or in fact related to ESA dose. Finally, our sample size was modest and further studies with larger numbers of patients should be performed to confirm our results.

## Conclusions

In conclusion, our study demonstrates that patients requiring high-dose ESA for treatment of CKD-related anemia are more likely to have increased levels of the pro-inflammatory biomarkers IL-6 and CRP. We also observed an association between higher dose ESA therapy and increased circulating endogenous sEpoR levels. Longitudinal studies of changes in sEpoR levels and cytokines with variations in ESA dosing and hemoglobin levels are warranted to confirm these findings.

## Competing interests

The authors declare that they have no competing interests.

## Authors' contributions

JI and LS created and designed the study. SB contributed to the collection and interpretation of the data. JI performed statistical analysis. UP contributed to the design of the study and to the interpretation of the data. MA contributed to the analysis and interpretation of the data. All authors have been involved in drafting the manuscript or revising it critically for important intellectual content and have read and approved the final manuscript.

## Pre-publication history

The pre-publication history for this paper can be accessed here:

http://www.biomedcentral.com/1471-2369/12/67/prepub

## Supplementary Material

Additional file 1 **Measurement of cytokines**. Detailed description of the techniques used to measure the reported cytokines. Click here for file

Additional file 2 **Measurement of soluble Epo receptor**. Detailed description of the techniques used to measure soluble Epo receptor. Click here for file
