# Supplementary material for: Association between high-dose erythropoiesis-stimulating agents, inflammatory biomarkers, and soluble erythropoietin receptors
Source: BMC Nephrol. 2011 Dec 12;12:67. doi: 10.1186/1471-2369-12-67 (PMC3254065; doi:10.1186/1471-2369-12-67)
Supplement: Additional file 1 — Measurement of cytokines. Detailed description of the techniques used to measure the reported cytokines. [file 1471-2369-12-67-S1.DOC]

**Additional File 1.**

**Measurement of cytokines**

Thermo Scientific SearchLight Protein Array Technology is a multiplexing sandwich-ELISA system based on chemiluminescent detection of analytes whose respective capture-antibodies are spotted in arrays within each well of a 96-well microplate. Protein arrays were created by spotting 9 different captured antibodies in each well of a 96-well plate using piezoelectric printing technology. Samples were added to the wells of the plate, resulting in the capture of appropriate target proteins by the arrayed antibodies. Then biotinylated antibodies were added that specifically bound to the captured proteins. Finally, an incubation step with streptavidin-horseradish peroxidase conjugate, followed by addition of SuperSignal ELISA Femto Chemiluminescent Substrate, resulted in the generation of chemiluminescence at spots where target proteins were captured. The entire plate was then imaged using a CCD imaging system to capture the chemiluminescent signal from each spot within each well. The concentration of each analyte in the array was quantified by comparing the spot intensities for each unknown sample to the corresponding standard curves calculated from the standard sample results by the SearchLight Array Analyst Software. Individual analytes were identified by the position of each specific capture antibody within the well. Standard curves, raw data and final pg/ml concentrations for each analyte and each sample were reviewed in the array software and exported to Microsoft Excel Software.
