# Supplementary material for: Association between high-dose erythropoiesis-stimulating agents, inflammatory biomarkers, and soluble erythropoietin receptors
Source: BMC Nephrol. 2011 Dec 12;12:67. doi: 10.1186/1471-2369-12-67 (PMC3254065; doi:10.1186/1471-2369-12-67)
Supplement: Additional file 2 — Measurement of soluble Epo receptor. Detailed description of the techniques used to measure soluble Epo receptor. [file 1471-2369-12-67-S2.DOCX]

**Additional File 2.**

**Measurement of soluble Epo receptor**

Briefly, polystyrene plates (R&D Systems, Cat DY990) were coated overnight at room temperature with mouse anti-human Epo R capture antibody (available for R&D). Plates were washed and then blocked with 1% bovine serum albumin (BSA) in phosphate buffered saline (PBS), 300 uL per well for 1 hour at room temperature.  Plates were washed again, then samples and standards added, 100 uL per well.  Samples were diluted 1:3 in 1% BSA in PBS.  The highest standard occurred at 4 ng/mL, with additional standards following 2-fold serial dilutions in 1% BSA, plus blank. The plates were then incubated 2 hrs at room temperature; washed; biotinylated mouse anti-human detection antibody, 100 uL per well, 90 ug/mL in 1% BSA, was then added; plates were incubated 2 hrs at room temperature; plates were washed; 100 uL streptavidin-HRP was added; plates were incubated 20 min; washed; 100 uL H2O2 substrate was added; incubated for 20 min; washed; and added 50 uL 2N H2SO4. The optical density of each well was then immediately determined using a microplate reader (Molecular Device M2e spectrophotometric plate reader) set to 450 nm.
